# Dopaminergic Plasticity in the Bilateral Hippocampus Following Threat Reversal in Humans

**DOI:** 10.1038/s41598-020-63977-7

**Published:** 2020-05-06

**Authors:** Jennifer I. Lissemore, Atsuko Nagano-Saito, Kelly Smart, Paul Gravel, Marco Leyton, Chawki Benkelfat

**Affiliations:** 1 Department of Psychiatry, McGill University, 1033 Pine Avenue West, Montreal, H3A 1A1 Quebec Canada; 2 Department of Neurology and Neurosurgery, McConnell Brain Imaging Center, Montreal Neurological Institute, McGill University, 3801 University St., Montreal, H3A 2B4 Quebec Canada

**Keywords:** Consolidation, Extinction, Fear conditioning, Hippocampus

## Abstract

When a cue no longer predicts a threat, a diminished ability to extinguish or reverse this association is thought to increase risk for stress-related disorders. Despite the clear clinical relevance, the mediating neurochemical mechanisms of threat reversal have received relatively little study. One neurotransmitter implicated in rodent research of changing associations with threat is dopamine. To study whether dopamine is involved in threat reversal in humans, we used high-resolution positron emission tomography (PET) coupled with ^18^F-fallypride. Twelve healthy volunteers (6 F/6 M) underwent three PET scans: (i) at baseline, (ii) following threat conditioning (the response to a cue associated with electric wrist shock), and (iii) following threat *reversal* (the response to the same cue now associated with safety). We observed moderate evidence of reduced dopamine D2/3 receptor availability, consistent with greater dopamine release, in the bilateral anterior hippocampus following threat reversal, in response to a safety cue that was previously associated with threat, as compared to both baseline and during exposure to the same cue prior to threat reversal. These findings offer the first preliminary evidence that the response to a previously threatening cue that has since become associated with safety involves dopaminergic neurotransmission within the hippocampus in healthy humans.

## Introduction

Pavlovian threat conditioning^1^, historically refered to as fear conditioning, is a classical learning paradigm in which a neutral cue is paired with an aversive stimulus, such that the cue can come to elicit many of the same effects as the threatening event^2^. If, at some point, the learned threat is no longer relevant, its expression can be inhibited by the learning of new associations^3^. This includes processes such as extinction learning, where the cue is presented repeatedly without the aversive stimulus, and reversal learning, where the conditioned response is extinguished and a new cue becomes associated with the aversive event. An important difference between these paradigms is that in threat extinction, threat is entirely absent during learning, whereas in threat reversal, threat remains present during learning, but associations with threat/safety are shifted to different cues. It has been proposed that an impaired ability to modify learned associations with threatening events can lead to maladaptive responses, increasing susceptibility to a range of psychiatric disorders, including post-traumatic stress disorder (PTSD), anxiety disorders, and obsessive-compulsive disorder (OCD)^4,5^.

Accumulating evidence suggests that threat extinction^6^ and reversal have overlapping neural correlates^7^ within mesocorticolimbic circuitry^5,8^. Using functional magnetic resonance imaging (fMRI), the inhibition of learned threat associations has been shown to increase blood flow in regions of mesocorticolimbic circuitry, including the ventromedial prefrontal cortex (vmPFC) and hippocampus^9,10^. The neurochemistry underlying these responses is poorly understood, but studies in laboratory animals implicate dopamine^11,12^, particularly within the prefrontal cortex^13^ and hippocampus^14^.

In humans, mesocorticolimbic dopamine plasticity has been associated with reward-related learning, including effects in the ventral tegmental area, ventral striatum, amygdala, hippocampus and medial prefrontal cortex^15–17^. Less is known about dopamine’s role in threat extinction and reversal in humans, but administration of the immediate dopamine precursor L-DOPA following threat extinction has been shown to enhance consolidation of the new safety memory in both mice and humans^18,19^.

To identify the brain regions where dopamine transmission is engaged during the recall of threat reversal in humans, the present study used positron emission tomography (PET) with ^18^F-fallypride, a highly selective, high affinity dopamine D2/3 receptor ligand^20–22^. We hypothesized that, compared to baseline (prior to threat conditioning), changes in ^18^F-fallypride binding would be observed within mesocorticolimbic circuitry both in response to a threat-associated cue and in response to the same cue following threat reversal.

## Results

### Participants

**Figure 1:**
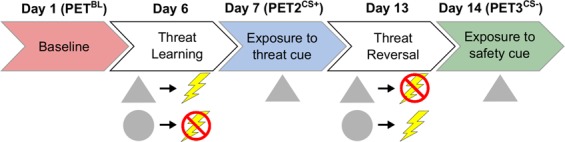
Study timeline. Timeline of the three PET scans and two stimulus pairing sessions.

Sixteen volunteers were enrolled in the study following initial screening procedures. One participant was excluded due to an inadequate autonomic response to the aversive stimulus during screening, one was excluded after the baseline PET scan (PET^BL^) due to a headache during this first PET scan, one withdrew after the MRI scan for unknown reasons, and one withdrew after the PET^BL^ session for unknown reasons. Therefore, a total of 12 volunteers (6 F/6 M, mean ± SD age = 24.1 ± 3.7 years) completed all study sessions (Fig. 1) and were included in the analyses.

### Subjective & autonomic indices of conditioned threat

All participants reported learning the correct associations between the neutral, conditioned stimuli (CS) and aversive, unconditioned stimulus (US), both immediately after the CS-US pairings and immediately prior to PET scanning. Accordingly, all participants reported associating “a little” to “moderate” anxiety with the conditioned cue paired with threat (CS+) following threat learning and prior to the second PET session (PET2^CS+^). Following threat reversal and prior to the third PET session (PET3^CS−^), 11 participants reported no anxiety associated with the same cue, which now predicted the absence of threat (new CS−). The 12^th^ participant indicated “a little” subjective anxiety associated with the new CS− prior to PET3^CS−^; although this participant reported the correct contingencies during reversal learning, a sensitivity analysis was performed with the participant’s data excluded.

The average intensity of electric shock rated to be at pain threshold was 35.4 V (range = 19–58 V). The anxiety scores associated with the CS+ for PET2^CS+^ and the new CS− (same cue) for PET3^CS−^ differed significantly (t_10_ = 9.8, *p* < 0.0001). Similarly, there was a significant main effect of PET session for the POMS composed-anxious subscale (F_2,22_ = 4.1, *p* = 0.03); post hoc analysis identified less anxiety in PET3^CS−^ than PET^BL^ (t_11_ = 2.3, *p* = 0.04) and PET2^CS+^ (t_11_ = 2.9, *p* = 0.01). Participants also reported feeling progressively less ‘sleepy’ across PET scans (VAS Sleepy subscale: F_2,22_ = 8.6, *p* = 0.002; PET^BL^ vs. PET3^CS−^ t_11_ = 4.1, *p* = 0.002; PET2^CS+^ vs. PET3^CS−^ *p* = 0.07), but significant changes in alertness across scans did not occur (VAS Alert subscale: F_2,22_ = 0.96, *p* = 0.4).

**Figure 2:**
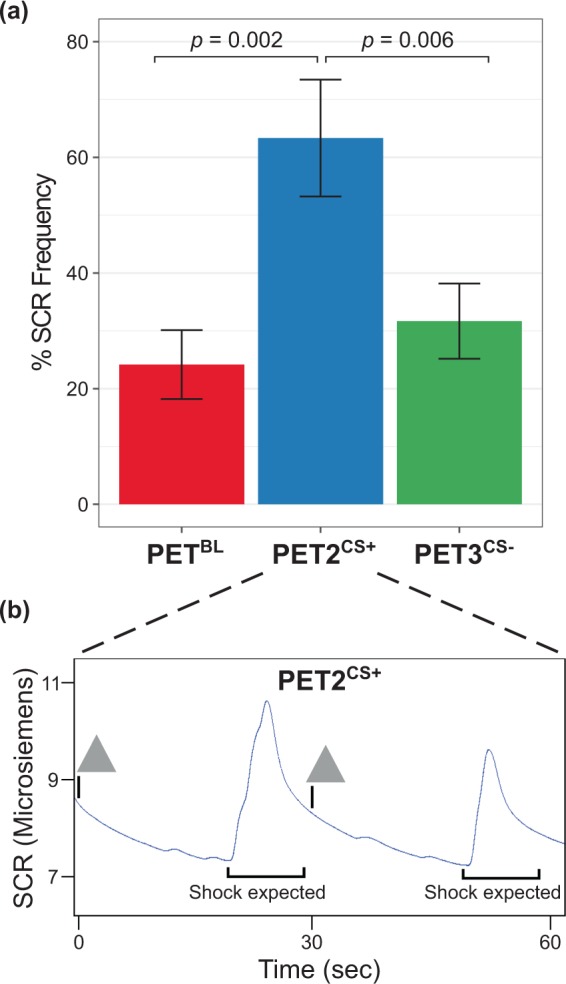
Autonomic evidence of conditioned responses to threat. (**a**) Percentage frequency of skin conductance responses (SCRs) in the first 10 trials of each PET scan. **(b)** An example from one participant of SCRs over time during PET2^CS+^ (in response to a cue associated with threat). Each trial was 30 s: the triangle indicates the onset of the 3 s conditioned stimulus, which was followed by a 20 s countdown, and a blank screen during which the participant expected to receive an aversive shock.

A significant main effect of PET session was found for the frequency of SCRs (F_2,22_ = 11.5, *p* < 0.001). Post hoc analyses revealed a significantly higher percentage frequency of SCRs in PET2^CS+^ (after presentation of the CS+, during anticipation of an aversive shock), as compared to both PET3^CS−^ (t_11_ = 3.41, *p* = 0.006) and PET^BL^ (t_11_ = 4.13, *p* = 0.002). By contrast, the percentage frequency of SCRs did not differ significantly between PET^BL^ and PET3^CS−^ (t_11_ = 1.08, *p* = 0.3), suggesting that the autonomic response to the former CS+ was effectively inhibited at PET3^CS−^ (Fig. 2).

### ^18^F-Fallypride binding potential

The mean ± SD injected activity of ^18^F-fallypride was 173.16 ± 12.58 MBq (PET^BL^: 175.45 ± 11.75, PET2^CS+^: 170.2 ± 13.20, PET3^CS−^: 173.90 ± 13.29 MBq). The mean ± SD specific activity was 246.80 ± 199.64 GBq/µmol (PET^BL^: 216.29 ± 150.55, PET2^CS+^: 284.15 ± 288.07, PET3^CS−^: 239.95 ± 136.32 GBq/µmol), corresponding to an injected mass of 0.43 ± 0.38 µg (PET^BL^: 0.47 ± 0.37, PET2^CS+^: 0.49 ± 0.53, PET3^CS−^: 0.33 ± 0.14 µg). No significant within-subject differences in injected activity, specific activity, or injected mass were observed across PET sessions (injected activity: F_2,22_ = 1.14, *p* = 0.34; specific activity: F_2,22_ = 0.43, *p* = 0.65; injected mass: F_2,22_ = 1.26, *p* = 0.30).

**Figure 3:**
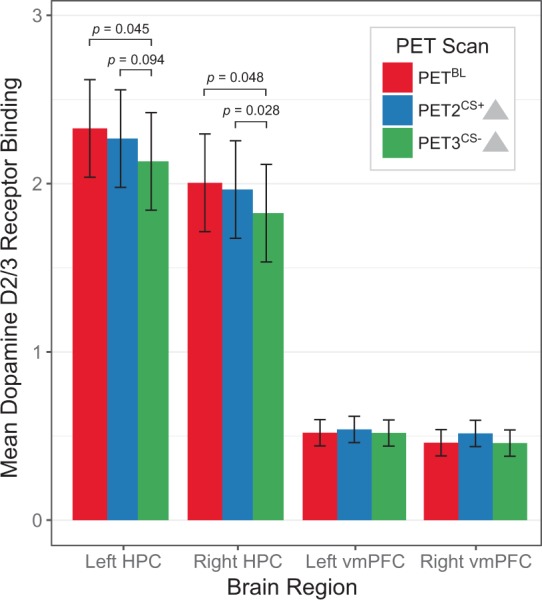
Dopamine receptor binding across PET sessions in the hippocampus (HPC) and ventromedial prefrontal cortex (vmPFC). Mean non-displaceable binding potential (BP_ND_) values are significantly lower in bilateral anterior hippocampus (HPC), but not in the vmPFC, in response to the updated safety cue following threat reversal (PET3^CS−^), as compared to baseline (PET^BL^) and in response to the same cue prior to threat reversal (PET2^CS+^). The observed decrease in BP_ND_ between scans is consistent with an increase in dopamine release. Error bars represent 95% confidence intervals.

Descriptive and linear mixed model statistics for each a priori-hypothesized ROI are summarized in Table 1. We found a significant main effect of PET session in the anterior hippocampus (F_2,55_ = 3.5, *p* = 0.037), with no significant hemisphere × session interaction (F_2,55_ = 0.01, *p* = 0.99). Post hoc analysis attributed the main effect of session to lower non-displaceable binding potential (BP_ND_) in PET3^CS−^ in the bilateral hippocampus, as compared to both PET^BL^ (t_11_ = 2.43, *p* = 0.033, *d*_Cohen_ = 0.70, mean [95% CI] change = −0.19 [−0.36, −0.02]) and PET2^CS+^ (t_11_ = 2.38, *p* = 0.037, *d*_Cohen_ = 0.69, mean [95% CI] change = −0.14 [−0.27, −0.01]). Exploring the laterality of the observed finding, BP_ND_ in PET3^CS−^ was significantly reduced in the right hippocampus, as compared to both PET^BL^ (t_11_ = 2.23, *p* = 0.048, *d*_Cohen_ = 0.64, mean [95% CI] change = −0.18 [−0.36, −0.002]) and PET2^CS+^ (t_11_ = 2.52, *p* = 0.028, *d*_Cohen_ = 0.72, mean [95% CI] change = −0.14 [−0.26, −0.02]), and in the left hippocampus, as compared to PET^BL^ (t_11_ = 2.27, *p* = 0.045, *d*_Cohen_ = 0.66, mean [95% CI] change = −0.20 [−0.39, −0.01]) (Fig. 3). A similar trend-level reduction was observed in left hippocampal BP_ND_ in PET3^CS−^ compared to PET2^CS+^ (t_11_ = 2.43, *p* = 0.094, *d*_Cohen_ = 0.70, mean [95% CI] change = −0.14 [−0.30, 0.03]). The observed decreases in anterior hippocampus BP_ND_ likely reflect increases in regional dopamine release during exposure to the updated safety cue following threat reversal, compared to both baseline and during exposure to the same conditioned cue prior to threat reversal. A sensitivity analysis excluding the data from one participant who reported reduced, but not totally absent anxiety in response to the new CS− prior to PET3^CS−^, yielded similar findings of decreased BP_ND_ in the bilateral hippocampus in response to the new safety cue (new CS−) following threat reversal (PET^BL^ vs. PET3^CS−^, t_10_ = 2.4, *p* = 0.035; PET2^CS+^ vs. PET3^CS−^, t_10_ = 2.5, *p* = 0.031).

**Table 1:** Descriptive and linear mixed model statistics for ^18^F-fallypride BP_ND_ across PET sessions. Mean ± SD BP_ND_ shown. VTA = ventral tegmental area, vmPFC = ventromedial prefrontal cortex.

Region of Interest (bilateral)	Non-Displaceable Binding Potential (BP_ND_)			Linear Mixed Model Statistics	
	Baseline (PET^BL^)	Response to threat cue (PET2^CS+^)	Response to safety cue (PET3^CS−^)	Main effect of session	Session × hemisphere interaction
Hippocampus	2.17 ± 0.56	2.14 ± 0.46	1.98 ± 0.36	F_2,55_ = 3.5 *p* = 0.037	F_2,55_ = 0.01 *p* = 0.99
Amygdala	3.62 ± 1.11	3.44 ± 0.69	3.25 ± 0.64	F_2,55_ = 2.9 *p* = 0.064	F_2,55_ = 0.05 *p* = 0.95
vmPFC	0.49 ± 0.12	0.53 ± 0.12	0.49 ± 0.14	F_2,55_ = 2.0 *p* = 0.14	F_2,55_ = 0.4 *p* = 0.65
Nucleus Accumbens	18.61 ± 4.09	18.29 ± 2.65	17.54 ± 3.05	F_2,55_ = 2.5 *p* = 0.093	F_2,55_ = 0.003 *p* = 0.99
VTA	2.11 ± 0.35	2.07 ± 0.31	2.06 ± 0.31	F_2,22_ = 0.3 *p* = 0.75	—

Complementing the linear mixed model and pairwise comparisons results, we observed moderate evidence in support of an effect of threat reversal on PET3^CS−^ BP_ND_ in the anterior hippocampus. A Bayes factor ANOVA with default priors showed that the PET session main effect model was preferred to the null model including hemisphere by a Bayes factor of 1.71, suggesting anecdotal evidence of an overall effect of PET session on BP_ND_ in the hippocampus. However, comparing BP_ND_ in PET3^CS−^ versus PET^BL^ and PET2^CS+^ separately, the data were 11.42 times and 8.63 times more likely under *H*_1_ than *H*_0_, respectively, indicating moderate evidence for a reduction in hippocampal BP_ND_ following threat reversal in particular. Bayes factors from the Bayesian analyses of a priori-hypothesized ROIs are reported in Table 2.

**Table 2:** Bayesian repeated measures ANOVA statistics. Bayes factors with percentage error for main effect and interaction models, and post hoc comparisons. All models are compared to the null model including hemisphere as a nuisance variable (*H*_0_; no effect). The posterior odds were corrected for multiple comparisons in the post hoc comparisons. Bayes factors >3 are bolded to indicate evidence for an effect (in favour of *H*_1_).

Region of Interest	Bayesian Statistics: Bayes Factor [error]		Post Hoc Comparisons: Bayes Factor [error]		
	PET Session	PET Session + PET Session*Hemisphere	PET^BL^ vs. PET2^CS+^	PET^BL^ vs. PET3^CS−^	PET2^CS+^ vs. PET3^CS−^
Hippocampus	1.72 [2.6%]	0.32 [2.4%]	0.26 [0.04%]	**11.42** [0.0004%]	**8.63** [0.0003%]
Amygdala	1.11 [2.4%]	0.23 [2.7%]	0.33 [0.04%]	**3.52** [0.0005%]	1.50 [0.007%]
vmPFC	0.55 [1.6%]	0.14 [2.9%]	0.63 [0.0001%]	0.22 [0.03%]	0.71 [0.0001%]
Nucleus Accumbens	0.81 [2.0%]	0.16 [5.4%]	0.26 [0.04%]	1.75 [0.005%]	1.44 [0.008%]
VTA	0.23 [0.7%]	—	0.32 [0.02%]	0.40 [0.02%]	0.29 [0.02%]

BP_ND_ values did not differ significantly between PET scans in the other a priori-hypothesized ROIs (Table 1). Accordingly, the Bayesian evidence for an effect of PET session on BP_ND_ in these ROIs was either inconclusive or in favor of the null hypothesis (Table 2). Although a reduction in amygdala BP_ND_ in PET3^CS−^ compared to PET^BL^ was favoured over the null hypothesis, the evidence for a reduction in amygdala D2/3 receptor availability following threat reversal was weak, and not replicated when comparing PET3^CS−^ with PET2^CS+^. Exploratory analyses also revealed that BP_ND_ values did not change significantly across PET scans in the anterior cingulate (PET session main effect: F_2,55_ = 0.72, *p* = 0.49; hemisphere × session interaction: F_2,55_ = 0.20, *p* = 0.82) or insula (PET session main effect: F_2,55_ = 0.55, *p* = 0.58; hemisphere × session interaction: F_2,55_ = 0.20, *p* = 0.82).

No significant correlations were observed between the change in SCR frequency and the change in regional BP_ND_ between PET scans. Similarly, no significant correlations were observed between changes in regional BP_ND_ and changes in subjective measures of mood or anxiety across scans. Of note, no significant correlations were observed between changes in BP_ND_ across PET scans in the hippocampus and changes in subjective measures of sleepiness across scans, suggesting that changes in levels of sleepiness did not account for the ^18^F-fallypride binding results reported here.

## Discussion

To our knowledge, the current study is the first investigation of dopamine release in humans following threat conditioning and reversal. We observed a significant and internally replicated decrease in dopamine D2/3 receptor availability in the bilateral hippocampus in response to a safety cue that had been previously associated with threat, as compared to both baseline and the response to the same cue prior to threat reversal. Bayesian analysis showed moderate evidence in favour of this effect. Evidence of decreased D2/3 receptor binding following threat reversal was also observed in the amygdala and nucleus accumbens, but the Bayesian evidence for these effects was inconclusive.

Both subjective reports and autonomic measurements during the PET scans confirmed that the presented conditioned cue was associated with electric shock following threat learning and with safety following threat reversal. Since the PET2^CS+^ and PET3^CS−^ sessions were identical, differences between scans in tracer binding likely reflect the changed significance of the conditioned cue. Since small reductions in ^18^F-fallypride binding are associated with large (>25-fold) increases in extracellular dopamine levels measured with microdialysis^23–25^, the bilateral decrease in hippocampal tracer binding in the PET3^CS−^ session is consistent with increased hippocampal dopamine release following threat reversal. Together, these findings constitute preliminary evidence that dopaminergic plasticity within the bilateral anterior hippocampus plays a role in safety signaling following the flexible updating of associations with threat.

Our findings in the bilateral hippocampus are consistent with past studies of learned responses to safety cues. An fMRI study in humans found that the hippocampus was activated in response to an extinguished threat cue, as compared to an unextinguished threat cue, and this activation correlated with the magnitude of extinction memory^10^. More recently, the conditioned inhibition of threat responding was found to activate neuronal subpopulations within the ventral/anterior hippocampus in both mice and humans^26^. A meta-analysis of fMRI studies suggests that these effects are relatively robust with significantly increased activity seen in the prefrontal cortex and anterior hippocampus in response to an extinguished/safety cue, as compared to an unextinguished/threat-associated cue^27^, similar to the findings from an earlier meta-analysis^28^. Studies in rodents suggest that these effects reflect causal mechanisms. Inactivation of the ventral hippocampus prior to extinction learning impairs extinction memory in rats^29^. Additionally, Pollak *et al*. showed that the ablation of hippocampal neurogenesis impairs learned safety in mice, and the systemic administration of dopamine agonists/antagonists alters the recall of learned safety^30^. More generally, the hippocampus may enable similar yet distinct associative memories to be stored as separate representations^31^. Of note, the meta-analyses did not identify consistent fMRI-measured activations in the amygdala in either threat learning or extinction recall^27,28^.

The specific involvement of hippocampal dopamine in the suppression of learned associations with threat has been less studied, but a recent study in rats found that the enhancement of threat extinction through exposure to a novel environment is dependent on dopamine D1 receptors in the hippocampus^14^. Within the context of the associative memory literature, our findings suggest that dopaminergic plasticity within the hippocampus may be involved in associative memory processes that underlie the inhibition of learned associations with threat in humans.

The current findings are relevant to disorders in which the inhibition of learned associations with threat is impaired, and in which mesocorticolimbic regions, such as the hippocampus, show abnormalities. For example, there is evidence of impaired extinction recall in PTSD patients^4^, and within the same patients, recall of an extinction memory correlated with hippocampal activation^32^. An improved understanding of the mechanisms involved in safety signaling following threat reversal is important for the optimization of exposure therapy for these disorders.

Contrary to our hypotheses, we did not observe significant ^18^F-fallypride binding changes within the vmPFC, nor did exploratory analyses identify effects in the anterior cingulate or insula. Each of these regions has been implicated in different aspects of fear and threat-related learning^27^. Dopamine, however, might contribute to only some of these responses. Indeed, regionally-specific subgroups of dopamine neurons within mesocorticolimbic circuitry exhibit distinct responses to different types of events and cues^33,34^ and vmPFC dopamine depletions in the marmoset do not influence performance on a reversal learning task^35^. The specificity of the current findings to dopamine neurons that innervate the anterior hippocampus is in line with this body of literature. Nevertheless, the vmPFC Bayesian analyses did not conclusively favour the null hypothesis, and studies in rodents suggest that dopaminergic activity in the PFC influences some aspects of extinction memory^13^. Since the vmPFC and hippocampus are highly connected^36^, and stimulation of the vmPFC has been shown to increase hippocampal cell proliferation and memory^37^, future studies should employ tracers that may be more sensitive to neurotransmitter release in cortical regions, such as ^11^C-FLB 457^38^.

The current study has limitations to consider. First, the sample size is modest due to the nature of PET imaging in general and demands of the present study in particular (e.g., >9 hours of PET scanning per participant). However, to our knowledge, this is the largest PET study reported to date on the inhibition of learned associations with threat in humans. We therefore consider the findings reported here thought provoking, yet requiring replication. Second, it is important to note that the findings were not corrected for multiple comparisons, however, the hippocampal dopamine response was observed bilaterally and Bayesian analyses indicated moderate evidence in favour of this result. Third, no shocks were administered during the second scanning session (PET2^CS+^), which constitutes both a strength and a limitation. Although the study design avoids the confound of administering an aversive stimulus during PET scanning, by repeatedly presenting the CS+ in the absence of shock, it is likely that the measured PET signal reflects a combination of conditioned threat and extinction learning or prediction error (whereby a shock is expected but does not occur). This is a confound inherent to all experimental designs that measure the response to the CS+ presented alone and might account for why compelling evidence of dopamine release in the PET2^CS+^ session was not seen. Fourth, without a separate control group, the effect of scan order on the ^18^F-fallypride signal cannot be ruled out, but ^18^F-fallypride BP_ND_ values show good test-retest reliability making this less likely^39,40^. Finally, there are inherent limitations to the use of ^18^F-fallypride. Although ^18^F-fallypride provides a reliable signal in brain regions with dopamine receptor densities that are lower than in the striatum, such as the hippocampus and amygdala, ^18^F-fallypride is not the optimal tracer for studying changes in striatal dopamine receptor availability; both ^11^C-raclopride and ^11^C-PHNO are considered superior for this purpose^20,41^. Decreases in ^18^F-fallypride binding observed are typically interpreted to reflect increased dopamine release, though it is possible that they reflect other forms of dopaminergic plasticity (including receptor internalization and trafficking, or changes in receptor conformational state).

In summary, despite its clinical relevance, threat reversal has been less extensively studied than other aspects of threat learning. The importance of dopamine in threat reversal remains particularly understudied. The present study provides the first preliminary evidence that dopaminergic activity within the anterior hippocampus is important for safety signaling following threat reversal in healthy humans. It also raises the possibility that abnormal hippocampal dopaminergic plasticity might play a role in psychiatric disorders characterized by a perseveration of responses to stimuli that are no longer threatening, such as PTSD, anxiety disorders and OCD.

## Methods

### Overview

The study entailed five test days including three PET scans. First, a baseline PET scan (PET^BL^) was performed while participants were presented with a white screen; no other stimuli were presented, and participants were instructed to relax with their eyes open. Prior to threat conditioning, participants also underwent an anatomical magnetic resonance imaging (MRI) scan for co-registration with PET. Approximately one week after PET^BL^, participants learned to associate a neutral visual cue with threat during the first stimulus pairing session (described below). One business day later, the second PET scan (PET2^CS+^) was performed, during which the conditioned cue associated with threat (CS+) was presented alone, without the aversive stimulus. Approximately one week after PET2^CS+^, a threat reversal paradigm was performed. One business day later, participants underwent the 3^rd^ and final PET measurement (PET3^CS−^), which was performed in an identical manner to PET2^CS+^, but the presented conditioned cue now predicted the absence of threat (new CS−). Data acquired during the third PET session reflect the response to the updated safety cue following threat reversal (Fig. 1).

### Participants

Healthy, right-handed volunteers aged 20–40 years were recruited using online advertisements on university websites. After a brief telephone screening, individuals who tentatively met the inclusion criteria underwent an in-person interview with the Structured Clinical Interview for DSM-IV Axis I Disorders (SCID)^42^, an electrocardiogram, blood work, a urine toxicology/pregnancy test, and a routine physical exam performed by a physician. Lastly, to verify that participants showed an adequate autonomic response to the aversive stimulus in the PET environment, baseline skin conductance and heart rate were first recorded during a 3-min rest period. Inclusion in the study required a >10% change in skin conductance and/or >1 SD change in heart rate from mean baseline values soon after mild electrical stimulation of the wrist. Exclusion criteria included a current or past Axis I disorder, family history of an Axis I disorder, serious physical illness, chronic medication use, regular tobacco (>5 cigarettes/day) and/or occasional cannabis use (>twice/month), as well as any counter-indications to MRI or PET. During screening, participants were familiarized with the PET room and scanner in order to minimize the effect of novelty during PET^BL^.

The study was carried out in accordance with the Declaration of Helsinki, and was approved by the Research Ethics Board of the Montreal Neurological Institute. All participants provided written, informed consent.

### Associative learning paradigm

Stimulus pairing sessions took place in the PET scanner, without scanning taking place. The presentation of all stimuli was programmed using SuperLab 4.5 (Cedrus Corporation, San Pedro, CA). All visual stimuli were presented in video glasses (OEM EVG920D Video Eyewear; 640 × 480 resolution, virtual display equivalent to 80″ at 1 m with a 35° viewing angle), compatible with the bore of the PET scanner. Electric pulses of 50 ms were administered using a stimulating bar electrode (Biopac convex unshielded bar electrode EL351, with 2 tin electrodes, spaced 30 mm apart) secured over the ulnar nerve of the left wrist. Electrode leads were connected to a Biopac STM200 (Constant Voltage Stimulator – Unipolar Pulse). The stimulating bar electrode was secured to the participant’s wrist during all pairing sessions, and during PET2^CS+^ and PET3^CS−^ (the stimulator was inactive during scans).

Both the acquisition and reversal of learned threat involved a cue-dependent, trace conditioning paradigm with partial reinforcement. The neutral, conditioned stimuli (CS) consisted of a grey triangle and a grey circle of equal area. The aversive, unconditioned stimulus (US) consisted of a mild electric shock to the non-dominant wrist just below or about at “pain threshold” (described below in *Subjective and Autonomic Measurements*). Each participant’s pain threshold was established at the start of the study, and immediately prior to each pairing session. One of the neutral cues (CS+) was followed by the aversive US in 30% of CS+ trials, whereas the other cue (CS−) was never followed by the US. The shape that was first paired with shock was counterbalanced across participants. A pairing session involved 20 trials (10 CS+, 10 CS−, in pseudorandom order), where each trial consisted of a 3 s CS presentation, followed by a 20 s countdown, and a 7 s blank screen during which participants either did or did not receive a brief shock. The low contingency rate (30%, i.e. 3 out of 10 CS+ trials were paired with shock) was employed to take advantage of the higher stress response to unpredictable stressors, as compared to predictable stressors^43,44^. By performing pairing in the PET scanner, context remained constant for both associative learning and subsequent recall; there is evidence that consistent context facilitates the retrieval of associative memories^45^. Pairing and scanning sessions were separated by 24 hours to allow for optimal memory consolidation prior to scanning^46^, and to further avoid an aversive stimulus confound.

The same pairing procedure was used for threat reversal (20 trials; 10 CS+, 10 CS−), except that the CS-US contingencies were reversed; the cue previously paired with the US was no longer followed by a shock (the CS+ became the new CS−), whereas the previously neutral cue was paired with shock (the CS− became the new CS+). Participants were not informed of the stimulus contingencies prior to pairing sessions.

### Subjective anxiety and autonomic measurements

To determine the appropriate electric shock intensity, the subjective pain threshold for each participant was defined as a 3 on a Numerical Rating Scale (0 = No Sensation; 1 = Just Noticeable; 2 = Uncomfortable; 3 = Pain Threshold; 4 = Painful; 5 = Maximum Tolerable), and at least 20 on a visual analog scale (VAS) of pain (0 = No Pain; 100 = Extremely Painful)^47,48^. A contingency awareness questionnaire was administered immediately after each pairing session to assess which CS the participant associated with shock, and the subjective anxiety associated with each CS (1 = None; 2 = A Little; 3 = Moderate; 4 = Extreme). The same questionnaire was administered immediately before PET2^CS+^ and PET3^CS−^ in order to prime the CS-US associative memory.

Subjective ratings of mood, anxiety and alertness were collected immediately before, and 30 and 150 minutes into, each PET scan. The questionnaires included the Profile of Mood States (POMS)^49^, state-trait anxiety inventory (STAI-State)^50^, and Alertness VAS^51^. POMS scores on 6 bipolar scales (elated-depressed, composed-anxious, energetic-tired, agreeable-hostile, confident-unsure, clearheaded-confused) were transformed into population normalized t scores.

Electrodermal activity and heart rate were measured continuously as autonomic indices of conditioned threat using Ag/AgCl disposable electrodes on the middle phalanges of the right index and middle fingers, and on the left and right sides of the chest. Electrodermal activity was analyzed as the frequency of skin conductance responses (SCRs), which reflect phasic deflections in the electrical conductivity of the skin. SCR data were analyzed offline using AcqKnowledge software. To assess the effectiveness of the CS at inducing event-related SCRs, we calculated the number of trials in which a phasic SCR occurred during the 30 s CS-US interval^52,53^, using a threshold for SCR detection of a base to peak difference>3 SD of baseline skin conductance. Baseline skin conductance was calculated as the mean skin conductance level during the 2 s interval before the CS onset. To minimize the impact of SCR habituation^54^, we calculated the frequency of phasic SCRs that occurred in the first 10 trials of PET2^CS+^ (in response to the CS+) and PET3^CS−^ (in response to the new CS−), or during the same time intervals in PET^BL^ (non-specific SCRs occurring in the absence of stimuli), as a percentage frequency per 10 stimulus presentations ((SCR count / 10 trials) × 100%).

### PET and MRI acquisition

Prior to each PET scan, a urine toxicology screen for illicit drugs of abuse was performed (Triage, Biosite Diagnostics, San Diego, CA), as well as a urine pregnancy test in women. PET measurements were performed using a high-resolution research tomograph dedicated brain scanner (HRRT; CTI/Siemens, Knoxville, TN) in the late morning to early afternoon. Scan resolution was 2.3–3.4 mm full width at half maximum.

First, a 6-min transmission scan was performed for attenuation correction, followed by a bolus injection of ^18^F-fallypride through an i.v. catheter in the left arm vein. Each PET scan was 3 hours in duration, consisting of 90 minutes of dynamic acquisition scanning, followed by a 30-minute break and a final 60-minute dynamic acquisition scan. The following sequence of frame durations was used during dynamic scanning: 3 × 10 s, 5 × 30 s, 4 × 60 s, 4 × 120 s, 5 × 300 s, 5 × 600 s and 6 × 600 s.

**Figure 4:**
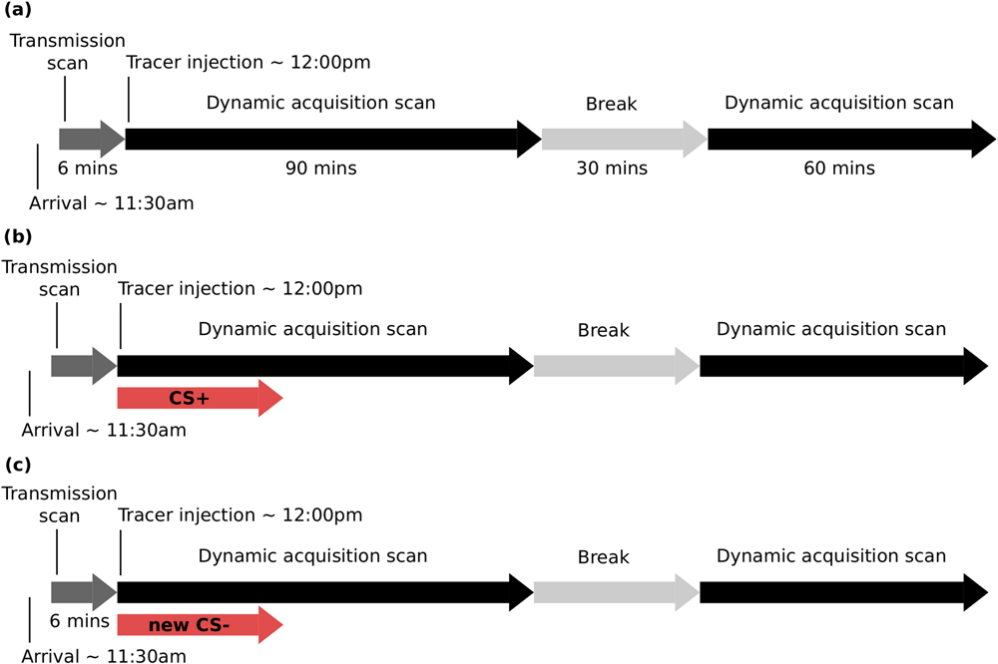
Timeline of each PET scan. (**a**) PET^BL^ with no stimulus presentations. **(b)** PET2^CS+^ with 60 presentations of the CS+ (neutral shape associated with shock). **(c)** PET3^CS−^ with 60 presentations of the new CS− (neutral shape no longer paired with shock).

In all PET sessions, participants were instructed to stay awake, keep their eyes open, and relax. During PET^BL^, participants were informed that no shocks would be delivered during the scan. Recording electrodes were set up, but the stimulating bar electrode was not. Following tracer injection in PET^BL^, participants were presented with a white screen. During both PET2^CS+^ and PET3^CS−^, participants were instructed that only one of the shapes from the previous pairing session would be presented during the scan. Following tracer injection in PET2^CS+^ and PET3^CS−^, the same conditioned stimulus (considered to be the CS+ during PET2^CS+^ and the new CS− during PET3^CS−^) was presented repeatedly during the first 30 minutes of scanning (60 trials per PET session). As in the stimulus pairing session, each trial consisted of a 3 s presentation of the CS, followed by a 20 s countdown, and a 7 s blank screen. The timeline of each PET scan is illustrated in Fig. 4.

MRI scans were conducted using a 3 T scanner equipped with a 32-channel head coil (Siemens TIM Trio Magnetom; Erlangen, Germany). A 9-minute T1-weighted anatomical MRI scan was performed (TR = 2300 ms; TE = 2.98 ms; flip angle = 9°; voxel size = 1.0 × 1.0 × 1.0 mm).

### PET and MRI data processing

PET data reconstruction was carried out using a maximum-likelihood expectation maximization iterative algorithm that corrects for scattered and random coincidences, attenuation, and detector-based non-uniformities^55^. PET frames were motion corrected using an automated algorithm^56^. The Simplified Reference Tissue Model (SRTM)^57^, with the basis functions method optimized for ^18^F-fallypride from ^11^C-raclopride studies^58^, was used to calculate BP_ND_ values at each voxel^20,59,60^. The cerebellar grey matter, which has minimal expression of D2/3 receptors, was used as a reference region. Following PET-MR co-registration and the transformation of the MRI scan and BP_ND_ map into MNI152 space, a 6-mm Gaussian filter was applied to the BP_ND_ map in order to reduce effects of anatomical variability.

Finally, regions of interest (ROIs) were defined bilaterally in the amygdala, anterior hippocampus, ventral tegmental area (VTA), nucleus accumbens and ventromedial prefrontal cortex (vmPFC). ROIs were also defined in the insula and anterior cingulate cortex for exploratory analyses based on recent fMRI evidence for the involvement of these regions in extinction recall^27^. ROI masks were created using the Wake Forest University (WFU) PickAtlas toolbox^61^ for SPM12, using the Automated Anatomical Labeling atlas (amygdala, hippocampus, vmPFC, anterior cingulate cortex and insula)^62^, the IBASPM 71 library (nucleus accumbens)^63^, and the VTA atlas from the Adcock lab^64^. Given that the ventral hippocampus in rodents, corresponding to the anterior hippocampus in humans, connects more densely to the amygdala^65,66^, receives stronger dopaminergic projections from the VTA^67^, and has been more widely implicated in trace conditioning, as compared to the dorsal hippocampus^68,69^, the relatively large automatically-segmented hippocampal ROI mask was manually reduced to include only anterior hippocampus. All ROIs were checked against individual MRI scans and adjusted manually if necessary. Mean BP_ND_ values were calculated bilaterally for each ROI from the BP_ND_ map in stereotaxic space, as well as by hemisphere for amygdala, hippocampus, nucleus accumbens and vmPFC ROIs. Given the relatively fast clearance of ^18^F-fallypride from limbic areas as well as cortex^25,39,70^, only the data from the first 90-minute scan were used for extra-striatal ROIs. A period of three hours is believed to be necessary to achieve transient equilibrium in the striatum^60^.

### Statistical analyses

We performed linear mixed-effects models for each ROI to assess changes in BP_ND_ across PET sessions by hemisphere, using a random effect of subject and fixed effects of PET session (3 timepoints: PET^BL^, PET2^CS+^, PET3^CS−^) and hemisphere (left, right). For bilateral VTA BP_ND_, injected dose, injected mass and specific activity of ^18^F-fallypride, as well as mood, anxiety and autonomic measures, linear mixed models were performed including subject as a random effect and PET session as a fixed effect. Planned pairwise comparisons consisted of two-tailed paired t-tests. Exact p-values are reported, uncorrected for multiple comparisons. The distributions of the residuals were checked using histograms and Q-Q plots, and the presence of influential outliers was evaluated using Cook’s distance. Pearson correlations were also performed between changes in BP_ND_ between PET sessions and changes in subjective and autonomic measures between PET sessions.

Lastly, we performed a Bayesian repeated measures ANOVA (two factors: PET session and hemisphere) for each ROI using JASP software^71–73^ in order to quantify the strength of evidence in favour of either the null hypothesis (*H*_0_: no effect of associative learning on regional dopamine release), or the alternative hypothesis (*H*_1_: an effect of associative learning on regional dopamine release). Bayes factors (BF_10_) were calculated for main effect and interaction models, including hemisphere as a nuisance variable. Post hoc comparisons were performed between PET sessions, with the posterior odds corrected for multiple comparisons^74^. A BF_10_ > 1 indicates evidence for an effect (*H*_1_), and a BF_10_ < 1 indicates evidence for no effect (*H*_0_). The strength of the evidence in favour of either hypothesis is considered to be of interest when BF_10_ is under 0.33 or over 3, otherwise the evidence is considered to be “anecdotal” and inconclusive^75^.

## Data Availability

The authors declare that the main data supporting the results in this study are available within the manuscript. The raw and analysed datasets generated during the study are available for research purposes from the corresponding authors on reasonable request.
